# Purification and characterization of bioactive compounds extracted from *Suaeda maritima* leaf and its impact on pathogenicity of *Pseudomonas aeruginosa* in *Catla catla* fingerlings

**DOI:** 10.1186/s13568-021-01295-5

**Published:** 2021-10-08

**Authors:** G. Beulah, D. Divya, N. S. Sampath Kumar, M. V. N. Sravya, K. Govinda Rao, Anjani Devi Chintagunta, G. Divya, S. Hari Chandana, B. D. Blessy, G. Simhachalam

**Affiliations:** 1 Department of Zoology and Aquaculture, Acharya Nagarjuna University, Guntur, Andhra Pradesh India 522510; 2 Department of Biotechnology, Vignan’s Foundation for Science, Technology and Research, Vadlamudi, Andhra Pradesh India 522213

**Keywords:** Aquaculture, *Catla catla*, *S. maritima*, Antibacterial activity, Antioxidant activity, In vivo

## Abstract

Incidence of various dreadful microbial infections and the development of antibiotic resistance by infection causative microbes are the main reasons for reducing aquaculture productivity. Hence, there is an immense need for the discovery of alternative and efficient treatment for quick recovery of diseased fishes. In the present study, *Suaeda maritima* leaf extracts (hexane, diethyl ether, ethanol, and water) were screened for in vitro and in vivo antibacterial and antioxidant activities. Out of all the four extracts, ethanolic extract showed highest antibacterial activity against *S. aureus* (4.9 ± 1.3 mm), *B. subtilis* (1.6 ± 0.3 mm), *K. pneumoniae* (4.2 ± 1.8 mm), and *P. aeruginosa* (4.1 ± 1.2 mm). Similarly, antioxidant activity was also higher for ethanolic extract (500 µg/mL) based on DPPH radical scavenging ability (71.6 ± 1.4%) and reducing potential (149 μg/mL) assays. Further, ethanolic extract was purified consecutively via column chromatography and preparative TLC where an active fraction was selected based on highest antibacterial (10.1 ± 1.4 mm) and antioxidant properties (82.3 ± 2.8%). Active fraction was loaded onto mass spectroscopy and identified the presence of four active constituents such as 1,2,9,10-tetramethoxy-6-methyl-5,6,6a,7-tetrahydro-4H-dibenzo[de,g]quinolin-3-yl) methanol; 3',7-Dimethoxy-3-hydroxyflavone; Saponin and (19R)9acetyl19hydroxy10,14dimethyl20oxopentacyclo[11.8.0.0 < 2,10 > .0 < 4,9 > .0 < 14,19 >]henicos-17-yl-acetate. Besides, in vivo studies were conducted on *Catla catla* fingerlings infected with *P. aeruginosa* under laboratory conditions. The fingerlings were segregated into 5 groups, among which group 4 and 5 were treated with crude and purified extracts. Both the extracts were efficient in treating infected fingerlings and recorded 100% survival rate which is even better than group-3 treated with a synthetic antibiotic (77%). Hence, *S. maritima* leaf extract can be considered as a possible alternative medicine in aquaculture.

## Introduction

Fish and other fish products from aquaculture represent a very valuable source of protein and essential micronutrients for balanced nutrition and good health for humankind. It is cheap and easily digestible animal protein which constitutes a significant share not only in global food basket but also in economy (Baldissera et al. 2017; Singh et al. 2020). Enormous increase in fish consumption is expected in mere future. Among the Indian major carps, *Catla catla* has high rate of consumption due to its low cost, rich source of proteins, polyunsaturated fatty acids, essential amino acids, vitamins and minerals (Sheikh et al. 2017; Kumar et al. 2019a, b). Increase in the demand has compelled aqua culturists to exercise intensification of culture system to increase production and profit on one end but on the other end pose stress to fishes (Kumar et al. 2019a, b; Rai et al. 2020). These stressful conditions results in the weakening of immune system and spread of virulent pathogens.

In most of the cases, fish disease outbreaks are highly contagious and spread very quickly in short span of time and lead to huge economic losses (Pękala 2018). Even though treating bacterial diseases with synthetic antibiotics is in practice, increasing risk of antibiotic resistant bacteria and accumulation of antibiotic residues in the environment and in fish tissues is challenging their frequent usage among farmers (ALsafah et al. 2017). As a remedy, natural therapy, which employs medicinal plants and their derivatives as ideal candidate is being used in aquaculture (Algammal et al. 2020; Kumar et al. 2021a, 2021b). Moreover, plants products from mangroves are considered as non-toxic, environmental friendly, inexpensive and biodegradable in nature (Saptiani et al.^2019^).

Mangroves are considered as biochemically unique due to their ability to produce a wide range of novel bioactive composites having enormous applications like control of bacterial, fungal and viral diseases in humans, animals and plants. Because of the presence of various bioactive compounds viz., steroids, triterpenes, saponins, flavonoids, alkaloids and tannins the mangrove plants are mostly used in therapeutics (Bhuela et al. 2020). For instance, mangrove plant extracts are playing a significant role in the treatment of various fish diseases because of their minimal side effects and therapeutic potential (Divya et al. 2020). About 1600 mangrove plant species are available in India and *Suaeda maritima* is one among them. It is used as an ethno medicine for curing various ailments such as hepatitis, bacterial infections, chronic inflammation and oxidative stress diseases (Nayak et al. 2018). It is a salt marshy mangrove plant that belongs to the family Chenopodiaceae and subfamily Suaedoideae and popularly known as seep weeds/sea-blites due their existence in coastal salt-flats and tidal wetlands (Kumar et al. 2019a, b). Considering its unique properties and abundance, the main goal of the current study is to prepare leaf extracts from *S. maritima* and assess its antibacterial and antioxidant properties under in vitro conditions. Further, the in vivo study was conducted on *Pseudomonas aeruginosa* infected *Catla catla*, where the mangrove extract was employed as an alternative approach for existing synthetic drugs.

## Materials and methods

### Collection and processing of plant

The present study majorly concentrates upon the use of *Suaeda maritima* in treating fish bacterial infections. This plant was collected from Gilakaladindi mangrove fields located in Machilipatnam (16° 0' N latitude and 81° 10' E longitude, Andhra Pradesh, India). Identification and confirmation of the collected plant was done at Botanical Survey of India (BSI).The collected leaves were cleaned thoroughly under tap water to remove dirt and rinsed using double distilled water followed by cutting of leaves into small pieces. The plant material is shade dried, pulverized and the resultant powder was stored in an air tight container.

### Chemicals

Hexane, Diethyl ether, ethanol, ascorbic acid, sodium hydroxide, ferric chloride, chloroform, acetic anhydride, sulphuric acid, glacial acetic acid, methanol, formic acid, acetonitrile and Potato Dextrose Agar (PDA) media used in the present study were procured from Merck.

### Extraction and phytochemical screening

Four different solvents viz*.,* hexane, diethyl ether, ethanol and distilled water have been used for extracting the bioactive compounds present in the leaves of *S. maritima*. The plant sample and the solvents are mixed in 1: 10 (w/v) ratio in a Soxhlet apparatus for 6 h at suitable temperatures. The extracts were filtered through Whatman No. 1 filter paper and concentrated using rotary evaporator (Divya et al. 2020). The concentrated crude extracts were used for screening various phytochemicals such astannins, alkaloids, flavonoids, saponins, steroids, terpenoids, phenols, proteins, glycosides, anthraquinones, phytosterols and reducing sugars using standard protocols (Harborne 1984).

### Antibacterial activity

Antibacterial activity of all the four extracts against *Staphylococcus aureus* (MTCC737), *Klebsiella pneumoniae* (MTCC 3384), *Bacillus subtilis* (MTCC 441) and *Pseudomonas aeruginosa,* (MTCC 1688) was determined through agar well diffusion method. 100 µl of bacterial cultures (12 h) were inoculated on to the petri plate containing solidified medium and dried for 5 min. In order to perform well diffusion method, equidistant wells, each with 6 mm diameter were cut in the agar with the help of a cork-borer. Further, 40 μl of each extract was loaded into the wells and amikacin (20 μg/mL) was used as a positive control. The plates were incubated for 24 h at 37 °C to find out their antibacterial efficacy of the extract. The antibacterial activity was assessed by measuring the diameter of zone of inhibition around the well containing *S. maritima* leaf extract (Guntur et al. 2018).

### Antioxidant activity

Conversely, the antioxidant activity of *S. maritima* leaf extract was determined using DPPH radical scavenging assay and FRAP assay.

### DPPH radical scavenging activity

For carrying out DPPH (2,2-diphenyl-1-picryl-hydrazyl) radical absorbance assay, the reaction mixture (5 mL) was preparedwith DPPH solution at different concentrations of extract (31.25, 62.5, 125, 250, 500 μg/mL). The mixture was incubation in the dark for 30 min and absorbance was measured at 517 nm. Ascorbic acid was used as standard and blank was prepared using methanol (Shaheena et al. 2019). Further, DPPH radical scavenging activity was calculated by the following equation:

$$\% DPPH \,radical \,scavenging\, activity=\left[\frac{\left(A0-A1\right)}{A0}\right] \times 100$$

where *A*0 is the absorbance of the control reaction and A1 is the absorbance of the sample of the tested extracts.

Similarly, the IC_50_ value was calculated by interpolation of linear regression analysis. Percentage of free radical activity was plotted against concentration of antioxidant substance so as to obtain the IC_50_ value.

### Ferric reducing antioxidant power (FRAP) assay

The reagent was prepared in 300 mM acetate buffer by adding 10 mM 2,4,6-tri (2-pyridyl-s-triazine) (TPTZ) solution in 40 mM HCl and 20 mM FeCl_3_ solution in proportion of 10:1:1 (v/v), respectively. All the four extracts and standard ascorbic acid at varying concentrations (15.62, 31.25, 62.5, 125, 250 and 500 μl/mL) are added to FRAP reagent and incubated at 37 °C for 15 min. The absorbance was measured at 593 nm and the results were recorded as μg of ascorbic acid equivalents (AAE) per mL (Benzie and Strain 1999).

### Purification and characterization of *S. maritima* leaf extract

#### Purification of sample by column chromatography

Active extracts of *S. maritima* (20 g) was subjected to column chromatography on silica gel of 100–200 mesh size (Merck) packed in a cylindrical glass column. Sample was passed in the pre-packed column and eluted with mixture of hexane: chloroform as mobile phase to vary polarity to separate and collect fractions. Extract was fractionated via gradient separation of mobile phase and each elute was carefully collected and labelled for further analysis. All the fractions were pooled based on similarity in colour and tested for antibacterial and antioxidant activity. Based on activity specific fractions were further purified using preparative TLC.

### Preparative thin layer chromatography (TLC)

Considering the results obtained from antibacterial and antioxidant activity, active fraction of *S. maritima* leaf obtained from column chromatography was subjected to TLC for separation of active compounds. Preparative TLC plates were prepared by homogeneous mixing of 30 g of silica gel and small amount of calcium sulfate in 60 mL of distilled water and uniformly spreading it on glass plates (20 × 20 cm) with 250 μ thickness. The plates were kept undisturbed for 10 min at room temperature followed by 1 h at 105 °C in hot air oven and then placed in a desiccator for 2 h. TLC of *S. maritima* active fraction was performed in the lidded tank comprising Butanol: Acetone: Water (12:6:3) as solvent system after spotting the sample on prepared silica gel plate. The solvent in the tank was maintained up to 1 cm beneath the origin. Because of the capillary action, the solvent travels on the plate along with the sample and various components of the sample gets separated. Formation of the bands was observed under the UV light. The procedure was followed until good resolution was noticed and each band was carefully scrapped and assessed for their antibacterial and antioxidant activity.

### Characterization of phytochemicals by mass spectroscopy

Crude and partially purified extracts of *S. maritima* were loaded onto Agilent 1100 LC/MS System with separate Chemstation Rev.A.09.01 (1206) software. Each extract (20 μL) was injected along with the mobile phase (formic acid (0.1%) in water (50%) and acetonitrile (50%)) at a flow rate of 0.5 mL/min. The electrospray ionization was set in negative ionization mode in 60–200 V and capillary voltage at 4000 V. Nitrogen is used as nebulising gas at 350 °C and 30 psi with flow rate of 8–10 L/min (Shivani et al. 2020).

## In vivo studies

### Experimental design

Fingerlings of *Catla catla* weighing 10 ± 0.9 g and length of 8 ± 0.5 cm were procured from local hatchery and screened for any pathogenic infections. Fish fingerlings were acclimatized in the aerated laboratory tubs contained 40 L of freshwater (temperature 28 ± 2 °C and 12:12 L: D period) 10 days prior to experimentation. The fishes were fed twice in a day with commercial food and 30% of water was changed daily to reduce ammonia toxicity. Various water parameters such as temperature: 26.49 ± 0.13 °C, pH: 7.94 ± 0.04, electrical conductivity: 392.22 ± 0.62 S/cm, total dissolved solids: 279.33 ± 0.69 mg/L, dissolved oxygen: 6.44 ± 0.05 mg/L, total alkalinity: 204 ± 7.30 mg/L as CaCO_3_, total hardness: 180.44 ± 3.74 mg/L as CaCO_3_, orthophosphate: 0.03 ± 0.001 mg/L, ammonia–nitrogen: 1.66 ± 0.21 mg/L and nitrate-nitrogen: 0.21 ± 0.03 mg/L were maintained during experimental period.

After acclimatization, fish fingerlings were randomly divided into 5 groups of nine each in triplicate and classified as group 1 (control), group 2 (negative control), group 3 (positive control), group 4 (crude extract) and group 5 (partially purified extract). Fish fingerlings in all the groups were starved for 24 h before initiating challenge studies and later fed with pellet feed containing 1 mL of *Pseudomonas aureginosa* suspension (10^3^ CFU). Group 1 was fed with regular feed and feeding was carried in a similar fashion after 12 h of interval. After 24 h, treatment for group 3, 4 and 5 was initiated with amoxicillin, crude extract of *S. maritima* (25 mg/g of body weight) and partially purified extract of *S. maritima* (10 mg/g of body weight) respectively along with feed twice a day for 5 days. The dosage of crude and partially purified extracts was determined on the basis of pilot study (data not shown). The remaining feed was siphoned out before the next feeding. Fingerlings were examined cautiously for pathological lesions, behavioural changes and mortality throughout the experimentation period.

### Confirmation of *P. aeruginosa*

Bacteria from experimental moribund fish fingerlings was isolated and inoculated on an agar plate by spread plate technique to satisfy the Koch’s postulates. The isolated bacteria were identified morphologically through Gram's staining and biochemically through catalase, indole, methyl red, citrate utilization and gelatin hydrolysis tests.

### Catalase activity (CAT) and Superoxide dismutase activity (SOD)

Liver was carefully dissected from all the groups followed by homogenization in Tris–HCl buffer (15 mM, pH 7.4) and centrifuged at 2000×*g* for 10 min for measurement of antioxidant enzyme levels. The supernatant was used for estimation of protein (Bradford 1976), CAT (Aebi 1984) and SOD (McCord and Fridovich 1969) activity. Catalase activity was measured at 240 nm to note the consumption of H2O2/min in the presence of sample. SOD activity was measured by mixing the sample with EDTA and pyrogallol and observed for increase in absorbance at 420 nm. Both the activities were expressed as units per mg of protein.

### Statistical analyses

All the experiments in the present study were performed in triplicates and the results were expressed as mean ± standard deviation. The statistical analysis was performed using IBM SPSS statistics for windows, 20.0 software (IBM Corp., Armonk, N.Y., USA).

## Results

### Preliminary phytochemical screening

Preliminary screening of phytochemicals was carried out for the crude extracts of *Suaeda maritima* leaves prepared using hexane, diethyl ether, ethanol and water. Among these extracts, ethanol and aqueous extract recorded presence of more number of phytochemicals than remaining two extracts (Table 1). Presence of glycosides, cardiac glycosides and reducing sugars was noticed in all the four extracts and steroids were present in the extracts of hexane, ethanol and water extracts. Based on the presence of phytochemicals with enormous medicinal value ethanol and aqueous extracts were considered out of four extracts for assessment of antibacterial and antioxidant activity.

**Table 1:** Phytochemical Screening of various leaf extracts of *Suaeda maritima*

Phytochemicals	Hexane	Diethyl ether	Ethanol	Water
Tannins	–	–	+	+
Saponins	–	–	+	–
Flavonoids	–	–	+	+
Glycosides	+	+	+	+
Terpenoids	+	–	+	–
Steroids	+	–	+	+
Phenols	–	+	+	–
Proteins	–	–	–	+
Phytosterols	–	–	+	+
Anthraquinones	–	–	+	–
Cardiac glycosides	+	+	+	+
Reducing sugars	+	+	+	+

‘+’ indicates presence, ‘–’ indicates absence

### Antibacterial activity

The antibacterial activity of ethanol and water extracts of *S. maritima* leaves was determined against *Staphylococcus aureus*, *Bacillus subtilis*, *Klebsiella pneumoniae* and *Pseudomonas aeruginosa*. Both the extracts were successful in inhibiting the growth of tested bacteria but comparatively ethanol extract showed highest inhibitory zone against *Staphylococcus aureus* (4.9 ± 1.3), *Bacillus subtilis* (1.6 ± 0.3), *Klebsiella pneumoniae* (4.2 ± 1.8) and *Pseudomonas aeruginosa* (4.1 ± 1.2) and values were very close to the activity of synthetic antibiotic amikacin (Table 2).

**Table 2:** Antibacterial activity of ethanol and aqueous leaf extracts of *Suaeda maritima*

Samples	Zone of inhibition (mm)			
	*Staphylococcus aureus*	*Bacillus subtilis*	*Klebsiella pneumonia*	*Pseudomonas aeruginosa*
Amikacin	5.1 ± 0.4	1.9 ± 0.6	4.6 ± 0.1	4.3 ± 0.9
Ethanol extract	4.9 ± 1.3	1.6 ± 0.3	4.2 ± 1.8	4.1 ± 1.2
Aqueous extract	3.6 ± 1.7	1.1 ± 0.7	3.4 ± 1.9	3.7 ± 1.5

Mean ± Standard deviation; n = 3

### Antioxidant activity of *Suaeda maritima*

**Fig. 1:**
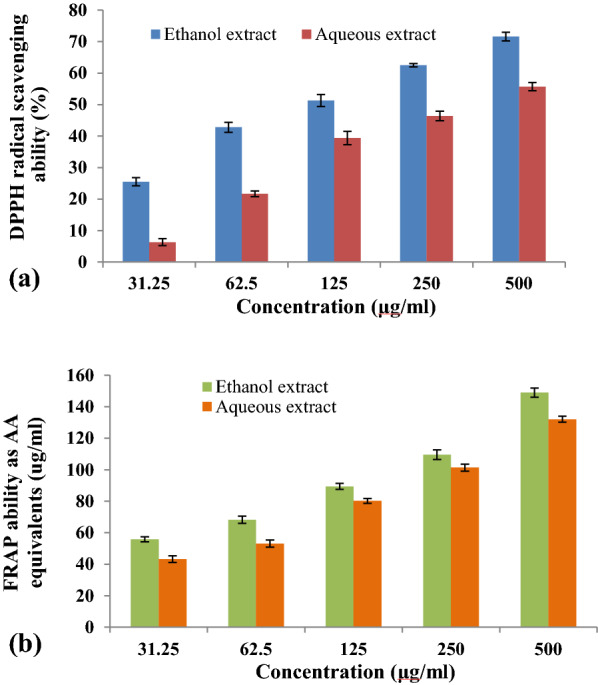
DPPH radical scavenging activity (**a**) and Ferric reducing antioxidant power ability (**b**) of *Suaeda maritima* leaf extracts

The antioxidant activities for the ethanol and aqueous extracts of *S. maritima* leaves at different concentration were studied through DPPH radical scavenging and FRAP assay. In DPPH radical scavenging assay, both the extracts were successful in donating proton and scavenging DPPH radicals (Fig. 1a). The IC50 value for ethanol and aqueous extracts was calculated based on plotting graph against concentration vs. percent of scavenging activity and determined as 123.48 µg/mL and 205.57 µg/mL respectively. The scavenging ability increases with increasing concentration and maximum ability was shown at 500 µg/mL as 71.6 ± 1.4% for ethanol extract and 55.7 ± 1.3% for aqueous leaf exact. On the other hand, the FRAP assay was conducted to determine the electron transfer ability of ethanol and aqueous extracts of *S. maritima* leaves and found their successful reduction of Fe^3+^ to Fe^2+^ by measuring the absorbance at 593 nm which confirms the antioxidant activity. Reducing potential was expressed in AAE μg/mL and gradually increased with increase in concentration as shown in Fig. (1b). Based on the present study, ethanol extract was found comparatively potent than aqueous leaf extracts with an AAE values of 149 and 132 μg/mL, respectively at 500 μg/mL concentration.

### Purification of ethanolic extract of *Suaeda maritima*

Ethanol extract of *S. maritima* was loaded onto the glass column packed with silica gel and allowed to interact with stationary phase using hexane as mobile phase. Molecules present in the *S. maritima* extract has interacted very well and spread throughout the column in couple of hours. Since the whole process was tested with various mobile phases and standardized, fractionation was carried out with single mixture i.e., hexane and chloroform with varying gradient. Total of eleven different ratios of mobile phase starting with 100% hexane to slowly shifting it towards 100% chloroform step by step has facilitated to separate the ethanolic extract of *S. maritima* into 12 fractions (Table 3). Based on diversity in color, all of them were pooled into four batches namely A, B, C & D. In view of applying purified ethanolic extract of *S. maritima* on *P. aeruginosa* infected fish fingerlings, same bacterium was used for testing antibacterial activity. Antibacterial activity estimated for four fractions confirmed its potential for fraction A and D with 8.3 ± 1.9 and 2.7 ± 0.3 mm zone of inhibition respectively (Table 3). On the other hand, three fractions A, B and C have shown good antioxidant potential and highest was recorded by fraction A with 72.6 ± 3.4% of DPPH radical inhibition making it as potent fraction for further purification.

**Table 3:** Fractions of *S. maritima* eluted using mobile phase (Hexane:Chloroform) at different gradients and their antibacterial and antioxidant activities

Mobile phase	Fractions	Code	Characteristics	*P. aeruginosa* (mm)	*DPPH radical scavenging activity (%)*
100:0	1–4	A	White	8.3 ± 1.9	72.6 ± 3.4
90:10	5–7	B	Light yellow	–	31.8 ± 1.9
80:20	–	–	–		
70:30	–	–	–		
60:40	–	–	–		
50:50	–	–	–		
40:60	–	–	–		
30:70	–	–	–		
20:80	–	–	–		
10:90	8–10	C	Yellow	–	63.8 ± 2.6
0:100	11–12	D	Brownish yellow	2.7 ± 0.3	–

Active fraction of *S. maritima* was further separated on preparative TLC by loading onto the manually prepared silica gel plate. Due to the capillary action of mobile phase, slowly compounds get separated into four long colourful streaks. Each streak was carefully scraped with sorbent layer from the plate and each of the streaks was tested for their antibacterial and antioxidant activity. As shown in Table 4, ES-2 has shown excellent antagonistic activity. Comparatively, all the streaks have shown better antioxidant activity than antibacterial activity and overall performance wise, both the properties were highly observed in ES-2 and same streak was used to confirm its potency in the in vivo conditions.

**Table 4:** Rf values and their corresponding bioactivites of active fraction of *S. maritima* after preparative TLC

TLC streaks	Rf value	Antibacterial activity against *P. aeruginosa* (mm)	DPPH radical scavenging activity (%)
ES-1	0.19	06.4 ± 0.9	52.4 ± 2.1
ES-2	0.23	10.1 ± 1.4	82.3 ± 2.8
ES-3	0.36	03.9 ± 0.8	27.2 ± 3.2
ES-4	0.49	02.5 ± 1.2	09.9 ± 1.3

### Phytochemicals profiling by mass spectroscopy

**Fig. 2:**
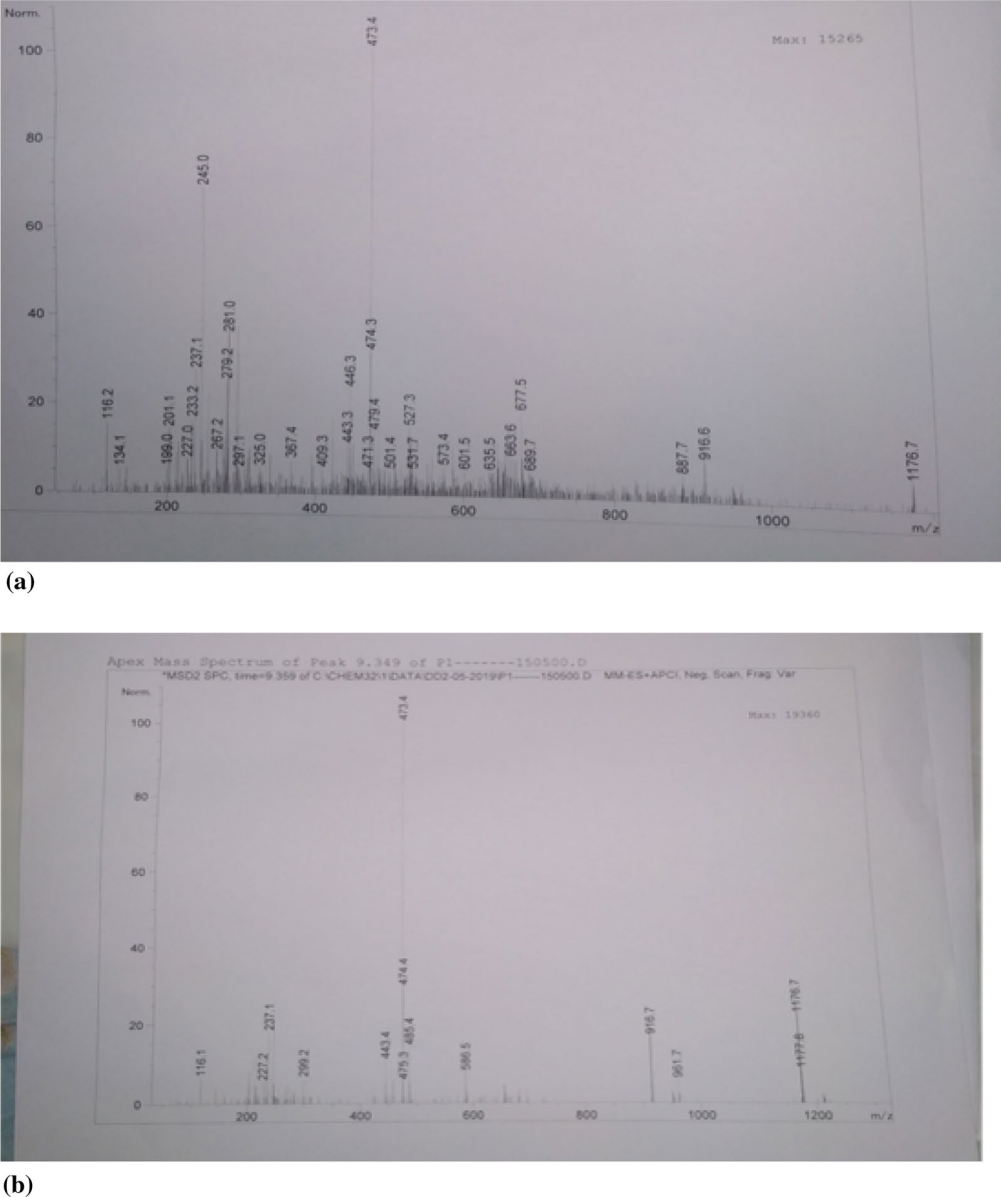
Phytochemicals profiling of **a** crude and **b** partially purified leaf extracts of *S. maritima*

Profiling of crude and purified ethanol leaf extracts of *S. maritima* by mass spectroscopy confirmed the presence of 12 and 3 bioactive molecules respectively (Table 5). These molecules were characterized based on the mass to charge ratio (m/z) of product or precursor ions and the same was compared with available literature and repository of reference compounds. Phytochemicals identified in the crude extract were Metolachlor-Morpholinone (233.2), 1,2,9,10-tetramethoxy-6-methyl-5,6,6a,7-tetrahydro-4H-dibenzo[de,g]quinolin-3-yl)methanol (237.1), Epicatechin (245.0), Lagochiline/Kaempferolacetyldisaccharides (281.0), Sclareol (325.0), 3(2′,4′-Dichlorophenyl)-4-phenylcoumarin (367.4), Haploside D (531.7), Tri-galloyl-hexoside I (635.5) and Icariin/ellagitannin (677.5) (Fig. 2). Upon loading the purified sample onto mass spectrometer, 4 molecules were identified1,2,9,10-tetramethoxy-6-methyl-5,6,6a,7-tetrahydro-4H-dibenzo[de,g]quinolin-3-yl)methanol; 3',7-Dimethoxy-3-hydroxyflavone; Saponin and (19R)9acetyl19hydroxy10,14dimethyl20oxopentacyclo [11.8.0.0 < 2, 10 > 0.0 < 4,9 > 0.0 < 14, 19 >]henicos-17-yl-acetate has a strong signature which must have influenced the biological activities of the extract.

**Table 5:** Phytochemicals profiling of crude and purified leaf extracts of *S. maritima*

m/z	Compounds	Structure	Crude extract	Purified fraction
233.2	Metolachlor-Morpholinone	C_14_H_19_NO_2_	Available	–
237.1	1,2,9,10-tetramethoxy-6-methyl-5,6,6a,7 -tetrahydro-4H-dibenzo[de,g]quinolin-3-yl) methanol	C_22_H_27_NO_5_	–	Available
245.0	Epicatechin	C_15_H_14_O_6_	Available	–
281.0	Lagochiline/Kaempferol acetyldisaccharides	C_20_H_36_O_5_	Available	–
297.1	3′,7-Dimethoxy-3-hydroxyflavone	C_17_H_14_O_5_	Available	Available
325.0	Sclareol	C_20_H_36_O_2_	Available	–
367.4	3(2′,4′-Dichlorophenyl)-4-phenylcoumarin	C_21_H_12_Cl_2_O_2_	Available	–
443.3	Saponin	C_19_H_19_N_7_O_6_	Available	Available
474.3	(19R)-9-acetyl-19-hydroxy-10,14-dimethyl-20-oxopentacyclo[11.8.0.0 < 2,10 > .0 < 4,9 > .0 < 14,19 >]henicos-17-yl acetate	C_27_H_40_O_5_	Available	Available
531.7	Haploside D	C_30_H_34_O_18_	Available	–
635.5	Tri-galloyl-hexoside I	C_27_H_24_O_18_	Available	–
677.5	Icariin/ ellagitannin	C_33_H_40_O_15_	Available	–

### In vivo studies of antibacterial and antioxidant activity

Leaf extract of *S. maritima* was assessed for its efficiency in recovering the *P. aeruginosa* infected *Catla catla* fingerlings. Symptoms such as change in the colour of skin, hemorrhages on abdomen, gills and fins were noticed within a few hours after infection in all the experimental animals. Fish fingerlings in group 2 (negative control group) started behaving abnormally with irregular movement and reached 100% mortality within 24 h of *P. aeruginosa* exposure. Dead fish fingerlings were collected to carry out post-mortem and found bloody fluid filled intestine with dispositional internal organs. Hemorrhages were noticed all over the intestine, liver and gills. To confirm the clinical symptoms recorded in these fingerlings, caused due to *P. aeruginosa* this bacterium was collected carefully and grown on the culture plate. As shown in the Table 6, both biochemical and morphological tests confirmed the isolated bacteria was *P. aeruginosa* there by fulfilling Koch’s postulation.

**Table 6:** Morphological and biochemical confirmatory tests for *P. aeruginosa*

S. no.	Characteristic	Result
1	Shape	Rod shaped
2	Gram staining	Gram negative
3	Catalase activity test	Positive
4	Gelatin hydrolysis test	Positive
5	Citrate utilization test	Positive
6	Indole test	Negative
7	Methyl red test	Negative

On the other hand, fishes belonging to group 3, 4 and 5 treated with synthetic antibiotic, crude and purified ethanol extract of *S. maritima*, has shown significant recovery after 12 h of infection. Group 3 had only 77% survival rate and total recovery was not achieved within 7 days of experimental period. Group 4 and 5 has 100% survival rate with variations in recovery time *i.e*., group 5 had maximum recovery within 4 days and group four had recovered on 7^th^ day due to the antibacterial potency of *S. maritima* extract. After the experimentation, liver from all the 5 groups was collected and assessed for the levels of antioxidant enzymes viz., CAT and SOD as shown in Table 7. Both the enzyme levels were reduced in group 2, as bacterial infection can initiate the free radical chain reaction leading to the depletion of antioxidant enzymes and in turn functioning of liver which can be recovered by treating the organism with natural antioxidants. As evident from the obtained results, CAT and SOD levels in group 4 and 5 are in close proximity to group 1 *i.e*. Control group.

**Table 7:** Catalase (CAT) and superoxide dismutase (SOD) activity in the liver of experimental *Catla catla* fingerlings expressed as units of enzyme activity/mg of protein

	Group 1	Group 2	Group 3	Group 4	Group 5
CAT	19.1 ± 1.1	10.5 ± 0.9	13.3 ± 0.8	16.4 ± 1.2	18.7 ± 0.3
SOD	12.5 ± 0.9	9.1 ± 0.6	10.6 ± 0.7	11.3 ± 0.9	12.1 ± 0.8

Mean ± standard deviation; n = 3

## Discussion

Fish disease in aquaculture plays a significant role in monetary loss by increasing cost for treatment and maintenance of their health. In natural systems, fishes are less prone to diseases because they are less crowded compared to culture systems (AL-Safah and AL-Faragi 2017). Intensification of aquaculture has been main reason for stress within the cultured fishes followed by outbreaks of various diseases. So, treating bacterial infection alone will not fulfil the purpose and moreover, multidrug resistance in bacteria has challenged the usage of synthetic/commercially available antibiotics (Sheikh et al. 2017; Chintagunta et al. 2020). In view of this, an attempt was made by preparing various solvent extracts of *Suaeda maritima* to identify a suitable and natural multi-potent molecule to treat fish diseases. All the four extracts have shown the presence of various secondary metabolites, where in majority of active constituents were present in ethanolic and aqueous extracts. Nayak et al. (2018) reported presence of saponins, terpenoids, tannins, alkaloids and steroids in the methanol leaf extracts of *Suaeda maritima*, besides their presence in other mangroves such as *Xylocarpus granatum*, *Avicennia marina*, *Salvador apersica* and *Avicennia officinalis* (Mulla and Chavan 2016). Similar to present study, ethanolic extract of *Sesuviumportula castrum* possesses phytochemicals like alkaloids, carbohydrates, cardiac glycosides, flavonoids, phenols, saponins, sterols, terpenoids, quinones, diterpenes, and resins contributed to its therapeutic value (Chintalapani et al. 2018).

In continuation, antibacterial assay and antioxidant activity assays were conducted on ethanolic and aqueous extracts based on the presence of good number of secondary metabolites and highest activity against *S. aureus, B. subtilis, K. pneumoniae,* and *P. aeruginosa* was shown by ethanol extract. Antibacterial properties of mangroves were well studied and species like *Hibiscus tiliaceus* showed antibacterial activity against *P. aeruginosa* (Andriani et al. 2017) and *E. agallocha* extract exhibited antibacterial activity against fish pathogenic bacteria such as *F. indicum, C. indologenes, C. gleum*, and *E. septic* (Razak et al. 2019). Dahibhate et al. (2020) evaluated the bactericidal activity of fourteen plants against pathogenic strains such as *S. aureus, S. epidermidis, E. faecalis* and *P. aeruginosa* proving the indubitable antibacterial activity of mangrove species. Similarly, highest antioxidant activity was also observed in ethanol extract based on the results obtained from DPPH radical scavenging and FRAP assays. Correspondingly, previous studies on ethanolic extract of *S. caseolaris* and *R. mucronata* have also shown similar potency (Dahibhate et al. 2020; Sadeer et al. 2019). Hence, the results obtained for antioxidant potency can be complimented with current results and existing literature.

Further, to enhance the bioactivity of ethanol extract, it was subjected to two step purification via column chromatography and preparative TLC consecutively. Purification and characterization of active molecules is mandatory as the real potential of a molecule is masked with impurities (Maria et al. 2005). Several techniques are available to achieve purification but, out of all, column chromatography and preparative TLC are unique, simple and cost effective because large amount of sample can be loaded and eluted at a time; which was once again proven from the present study. The antibacterial activity against *P. aeruginosa* was increased from crude to purified fraction from 4.1 ± 1.2 to 10.1 ± 1.4 mm. Both crude and purified fraction was loaded onto mass spectrometer to identify the active constituents responsible for biological activity. Crude extract shown 11 active compounds, which were known to exhibit antibacterial, antioxidant, anti-inflammatory and anticancer properties based on the literature (Chen and Chen 2013; Popova et al. 2020; Sobeh et al. 2019). Whereas, purified fraction showed 4 active metabolites out of which saponins serve as anti-feedants besides capable of acting antagonistically against microbes and fungi (Moses et al. 2014). Similarly, a strong flavonoid at 297.1 m/z identified as 3′,7-Dimethoxy-3-hydroxyflavone was reported by Jasril et al. (2003) to exhibit antioxidant and antitumor promoting properties.

Finally, in-vivo studies were conducted on *P. aeruginosa* infected *Catla catla* fingerlings because infections caused by bacteria are one of the major threats reported in intensification of aquaculture and *P. aeruginosa* is one among the top three bacteria that are responsible for high mortality of Indian major carps (Pękala 2018). *P. aeruginosa* is opportunistic and common species of micro biota capable of degrading fish and fish products if not treated with suitable agents (Algammal et al. 2020). Similar kind of adversity was also recorded in group-2, which acted as negative control and was not treated with synthetic antibiotic/ *Suaeda maritima* extracts. Autopsy on dead fishes was conducted and bacteria from these fishes was isolated, cultured and identified as *P. aeruginosa* to fulfil Koch’s postulates. Similar confirmatory tests were carried out by Thomas et al. (2014) and Algammal et al (2020) for confirmation of *P. aeruginosa* in *O. mossambicus*, *O. niloticus* and *C. gariepinus*. On the other hand, both crude and purified extracts of *Suaeda maritima* were efficient in treating the bacterial infection with 0% mortality. Such recovery during bacterial infection was reported earlier from *Avicennia marina* leaves extract in infected *Labeo rohita* (Kumar et al. 2019a, b) and *Thymus vulgaris* extract in *Cyprinus carpio* (AL-Safah and AL-Faragi 2017). Such antimicrobial potency of herbal extracts is not confined to treating fishes but also was successful on treating tiger prawn by *Rhizophora mucronata* and *Sonneratia alba* (Saptiani et al 2019).

Till date, numerous synthetic antibiotics are being used for treating diseases caused by infectious microbes. Eventually, these microbes are developing resistance against antibiotics and actively spreading dreadful infections responsible for huge loses in aquaculture. Thus, the application of synthetic drugs to treat various ailments need to be replaced with biological molecules. The leaf extract of *Suaeda maritima*, a mangrove plant, found to be rich in phytochemicals exhibiting antibacterial and antioxidant activities. In the present study, it is observed that crude and partially purified extracts were effective in recovering infected fishes within a short span of time. Thus, *Suaeda maritima* extract possesses healing ability which will be very much beneficial for the aquatic fauna and fish cultivators.

## Data Availability

The data that support the findings of this study are available on request from the corresponding author. The data are not publicly available due to privacy ethical restrictions.
